# Laser-Mediated Hemostasis for Older Patients Receiving Routine Dental Treatment

**DOI:** 10.3390/dj13070315

**Published:** 2025-07-11

**Authors:** Suwat Tanya, Saengsome Prajaneh, Piyachat Patcharanuchat, Sajee Sattayut

**Affiliations:** 1 Graduate School, Faculty of Dentistry, Khon Kaen University, Khon Kaen 40002, Thailand; suwat.tanya@kkumail.com; 2 Lasers in Dentistry Research Group, Khon Kaen University, Khon Kaen 40002, Thailand; sanpra@kku.ac.th (S.P.); piypat@kku.ac.th (P.P.); 3 Department of Oral Biomedical Sciences, Faculty of Dentistry, Khon Kaen University, Khon Kaen 40002, Thailand; 4 Department of Preventive Dentistry, Faculty of Dentistry, Khon Kaen University, Khon Kaen 40002, Thailand; 5 Department of Oral and Maxillofacial Surgery, Faculty of Dentistry, Khon Kaen University, Khon Kaen 40002, Thailand

**Keywords:** laser therapy, photocoagulation, photobiomodulation, blood coagulation, tooth extraction, scaling and root planing, postoperative bleeding

## Abstract

**Background/Objective:** Laser therapy has gained attention in dental practice to minimize bleeding and enhance blood clot formation. This study aimed to explore the utilization and to compare the clinical efficacy of laser-mediated hemostasis for older patients receiving routine dental treatment. **Methods:** A prospective observational study was conducted across research networks between October 2023 and August 2024, involving 60 patients aged 50 years and older (average = 63.35 years) at risk of postoperative bleeding following dental treatments. Additionally, laser therapy for hemostasis was selected and provided among calibrated operators. A single researcher performed data collection. Before statistical analysis, data verification and clinical assessment were conducted by the operators and researcher. A clinical cut-off for hemostasis was set at 5 min. Two diode laser machines were used namely, an 810 nm and dual wavelengths of 635 nm and 980 nm. **Results:** There were 94 extraction sockets, 28 procedures of scaling and root planing and 18 procedures of minor oral surgery. Combining laser ablating sulcular fiber and photobiomodulation initiating blood clot formation was a preferable hemostatic technique for extraction socket, while photobiomodulation alone was a preferred technique for soft tissue hemostasis (*p* < 0.001). All operators confirmed that 97.86 percent of bleeding events achieved more rapid hemostasis. 61.43 percent of bleeding events clinically achieved hemostasis within 5 min by using laser-mediated hemostasis alone (*p* = 0.092). Full recovery of the extraction socket was significantly observed during the 2- to 4-week follow-up period (*p* = 0.005). No clinical complications were reported. **Conclusions:** Laser-mediated hemostasis effectively reduced hemostatic duration, prevented postoperative bleeding and promoted wound healing in older patients undergoing routine dental treatment.

## 1. Introduction

Routine dental treatment performed in the older population is challenging due to an elevated risk of both perioperative and postoperative bleeding which is primarily associ- ated with medication-induced bleeding tendencies and severe gingival inflammation [1]. Although routine simple tooth extraction and scaling and root planing (SRP) were generally considered as low-risk procedures of bleeding in patients underwent oral antithrombotic therapy [2], evidence of severe postoperative bleeding following routine tooth extraction in older patients has still been reported [3,4]. While several studies have indicated that severe bleeding after SRP was not common [5,6,7], there were reports of postoperative bleeding [3] and even hypovolemic shock [8] in older patients resulting from severe gingival bleeding. In addition, poor periodontal health was high prevalent among older patients with diabetes, contributing to increase gingival inflammation and impair wound healing [9]. The high burden of comorbidities, the use of oral antithrombotic therapy and age-related factors that compromise the coagulation system further complicate bleeding risk and clinical management in the older population. These clinical challenges make it difficult to apply effective hemostatic measures after routine dental treatments, such as tooth extraction, SRP and minor oral surgery.

To address these challenges, the laser therapy method known as laser-mediated hemostasis has recently been implemented for routine tooth extraction in older patients across all levels of the healthcare system in Thailand [1,10,11,12]. This method utilizes high-intensity laser therapy (HILT) with either an 810 nm or 980 nm laser, operating at a power of 2.5 to 3 watts, and employing a 320 µm optical fiber to ablate the sulcular fibers during the soft tissue loosening process involved in the extraction. Following this, Photobiomodulation Therapy (PBMT) is applied to promote blood clot formation in the extraction socket. This can be done using either a 635 nm diode laser at 0.2 watts and 5.08 J/cm^2^ with an 8 mm biomodulation probe or an 810 nm diode laser at 0.8 watts and 5.93 J/cm^2^ with a 6 mm biomodulation probe. This combined approach effectively achieves clinical hemostasis in most extraction sockets within 5 min. A significant advantage of these techniques is their minimally invasive nature, which makes them easy for healthcare providers to use. Moreover, older patients have reported high levels of acceptance and satisfaction with laser therapy [12].

Although laser-mediated hemostasis has been successfully used in tooth extractions, there is still a lack of evidence regarding its application in other routine dental treatments such as SRP and minor oral surgeries, which can also lead to perioperative and postoperative bleeding. Attaining pressure hemostasis following SRP is also more challenging than in an extraction socket, as the latter is surrounded by bony walls that facilitate effective compression and clot stabilization. Severe gingival inflammation further complicates the hemostatic process. In deeper periodontal bleeding sites, particularly within the sulcus and interproximal areas, compression alone is inadequate [5,12].

The objectives of this study were to explore the utilization of laser-mediated hemostasis techniques and to compare the clinical efficacy of hemostasis achieved within 5 min. The procedures examined included tooth extraction, scaling and root planing (SRP), and minor oral surgery among older patients receiving treatment within the research networks of the Lasers in Dentistry Research Group (LDRG) at Khon Kaen University.

The null hypothesis stated that there would be no significant difference in the proportion of procedures achieving hemostasis within 5 min across the different dental treatments. In contrast, the alternative hypothesis proposed that the proportion of procedures achieving hemostasis within this time frame would differ significantly among the various dental treatments.

## 2. Materials and Methods

### 2.1. Study Design and Setting

This study was a prospective observational study. Data collection was performed between October 2023 to August 2024 at the research networks of the Lasers in Dentistry Research Group (LDRG), Khon Kaen University (KKU), Khon Kaen, Thailand: (i) Bua Ngoen Sub-district Health Promoting Hospital, Nam Phong District, Khon Kaen Province, (ii) Borabue Hospital, Borabue District, Mahasarakham Province and (iii) Orofacial Laserology Clinic, Department of Oral and Maxillofacial Surgery, Faculty of Dentistry, Khon Kaen University. Routine dental procedures and adjunctive laser-mediated hemostasis were selected and performed by experienced dentists, who were certified in orofacial laserology by LDRG, KKU. All dental procedures were part of routine treatments at each research site. The operators were educated and trained in laser safety, fundamental laser knowledge and laser-mediated hemostasis techniques. All operators had over five years of experience in utilizing laser therapy in a clinical setting. The data was randomly collected by a single researcher (ST) who visited the research sites where cases were receiving routine treatment.

The research project has been reviewed and approved by the Khon Kaen University Ethics Committee for Human Research based on the Declaration of Helsinki and the ICH good clinical practices guideline (HE 642211).

### 2.2. Sample Size Estimation

The sample estimation was based on a previous study reporting 64 percent of bleeding extraction sockets achieved clinical hemostasis within 5 min following laser-mediated hemostasis [12]. To compare the same clinical outcomes between the reference group (*p*_1_ = 0.64) and the comparison group (*p*_2_ = 0.14), presenting a difference proportion of 0.50, a sample size calculation was conducted using the standard formula for comparing two independent proportions:

$$n=\frac{{\left[Z_{1-\alpha /2}∙\sqrt{2\bar{p}\left(1-\bar{p}\right)}+Z_{1-\beta }∙\sqrt{p_{1}\left(1-p_{1}\right)+p_{2}\left(1-p_{2}\right)}\right]}^{2}}{{\left(p_{1}-p_{2}\right)}^{2}}$$

where $\bar{p}=\frac{p_{1}+p_{2}}{2},Z_{1-\alpha /2}=1.96$ for a two-sided significance level of 0.05, $Z_{1-\beta }=0.84$ for 80% power of test. The estimated sample size per group was approximately 14 procedures. Accounting for a potential 20% drop-out rate, the sample size was increased to 17 procedures per group.

### 2.3. Participant Recruitment

In each research network, patients were initially screened for eligibility by the operator and then purposively invited by the researcher (ST). All included patients provided written informed consent before participation.

Older patients eligible for this study were at least 50 years old, classified as American Society of Anesthesiologists (ASA) Physical Status I to IV, literate, and required routine dental treatments (routine tooth extraction, SRP or minor oral surgery), at risk of bleeding from medication-induced bleeding tendencies or local gingival inflammation assessed by the operator. These criteria were applied across all research networks. Exclusion criteria included hereditary bleeding disorders, a history of organ transplantation, allergies to local anesthesia and unwillingness to participate. Patients who required surgical removal of tooth extraction were withdrawn from the study. Additionally, those with severe systemic diseases or receiving oral antithrombotic therapy underwent a physician consultation prior to dental treatment.

### 2.4. The Procedure of Laser-Mediated Hemostasis

This study applied two techniques of laser-mediated hemostasis: HILT for sulcular fiber ablation and PBM for initiating blood clot formation [12]. Chosen laser therapies were based on the operator’s decision. Regarding the available laser machine, an 810 nm diode laser machine (PICASSO LITE, AMD Lasers, Indianapolis, IN, USA) was utilized at Bua Ngoen Sub-district Health Promoting Hospital and Borabue Hospital. A 635 and 980 nm diode laser machine (SmartM, Lasotronix, Piaseczno, Poland) was available at Orofacial Laserology Clinic, Department of Oral and Maxillofacial Surgery, Faculty of Dentistry, Khon Kaen University. All operators and patients adhere to laser safety measures and the compulsory use of protective eye protection during procedures.

#### 2.4.1. HILT Ablating Sulcular Fiber

**Figure 1:**
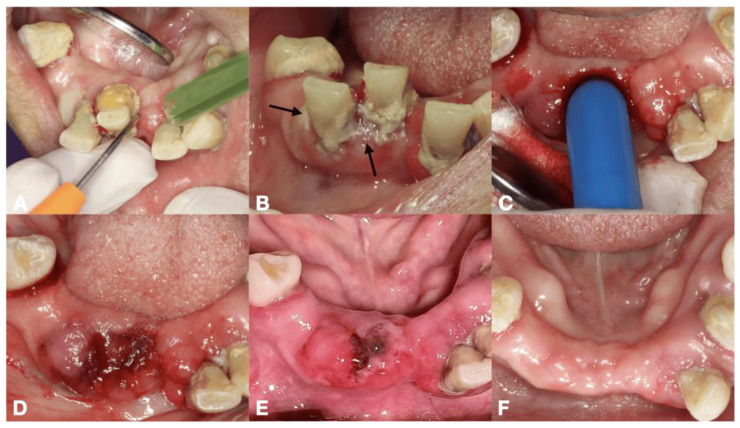
The procedures of laser-mediated hemostasis for tooth extraction in 68-year-old Thai female with hypertension and dyslipidemia, (**A**) HILT ablating sulcular fiber by using 810 nm diode laser at 2 W with optical fiber, (**B**) black arrows indicating a whitish coagulative area on gingivae, (**C**) PBM initiating blood clot formation by using 810 nm diode laser with a 6 mm-diameter biomodulation probe, (**D**) complete clot formation found within 5 min, (**E**) 1-day follow-up period presenting a coagulum with less swelling, minimal inflammation and no pain, (**F**) 4 weeks follow-up period indicating complete mucosal coverage of the edentulous ridge.

**Figure 2:**
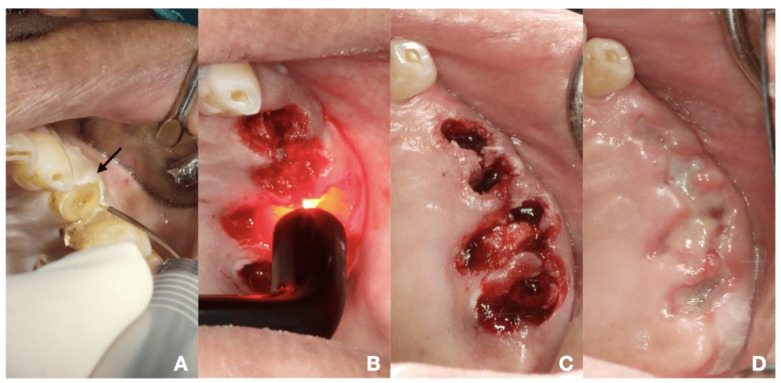
The procedures of laser-mediated hemostasis for tooth extraction in 64-year-old Thai male with coronary artery disease (triple vessel disease), hypertension, diabetes, history of stroke and ongoing dual antiplatelets therapy (clopidogrel 75 mg and aspirin 81 mg). (**A**) HILT ablating sulcular fiber by using 980 nm diode laser at 3 W with optical fiber, black arrow indicating a whitish coagulative area on gingivae, (**B**) PBM initiating blood clot formation by using 635 nm diode laser with 8 mm-diameter laser probe, (**C**) complete blood clot formation in all extraction sockets found within 5 to 15 min, (**D**) 7 days follow-up period indicating partial mucosal coverage and coagulum.

This technique aimed to ablate sulcular fibers and coagulate gingival tissue by moving a fiber optic up and down inside the gingival sulcus until a coagulative zone appeared in a whitish color. The insertion depth was approximately 2 to 3 mm from the marginal gingiva, avoiding periosteum destruction. The laser parameters were an 810 nm diode laser providing power of 2.5 W in continuous wave mode (PICASSO LITE, AMD Lasers, Indianapolis, IN, USA) or a 980 nm diode laser (SmartM, Lasotronix, Piaseczno, Poland) at a power of 3 W in continuous wave mode (Figure 1A and Figure 2A). The laser operating parameters were detailed in Table 1.

#### 2.4.2. PBM Initiating Blood Clot Formation

**Figure 3:**
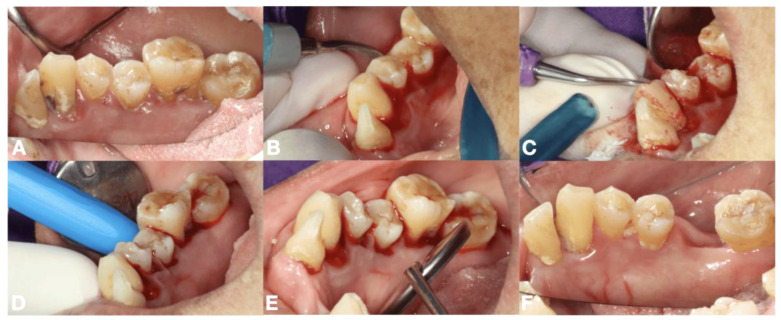
The procedures of laser-mediated hemostasis for SRP in 58-year-old Thai female with diabetes and hypertension, (**A**) untreated periodontal diseases with supra and subgingival calculus and inflamed gingivae, (**B**,**C**) SRP using ultrasonic scaler and hand instrumentation, (**D**) PBM for initiating blood clot using 810 nm diode laser with biomodulation probe over the bleeding gingival sulcus, (**E**) complete clot formation found within 10 min, (**F**) 4 weeks follow-up period indicating healthy periodontal tissue.

PBM initiating blood clot formation aimed to enhance the initial clot formation following tooth extraction, SRP or minor oral surgery. Regarding laser machine available, there were two laser parameters as follows: (i) an 810 nm diode laser (PICASSO LITE, AMD Lasers, Indianapolis, IN, USA), the pre-setting providing an actual power of 0.8 W, delivering an energy density of 5.93 J/cm^2^ in continuous wave mode via a 6 mm diameter biomodulation laser probe (Figure 1C and Figure 3D) and (ii) a 635 nm diode laser (SmartM, Lasotronix, Piaseczno, Poland) with a power output of 0.2 W in continuous wave mode, delivering an energy density of 5.08 J/cm^2^ with an 8 mm diameter laser probe were suggested (Figure 2B). PBM was irradiated over the bleeding extraction socket, bleeding gingival sulcus or soft tissue bleeding. The number of irradiations was based on clinical observation. The initial four sessions were applied [12].

### 2.5. Criteria for Evaluating Blood Clot Formation

The criteria were as follows: (i) active bleeding: obviously observed blood flow, (ii) sluggish oozing: observed blood flow and (iii) clot formation: no blood flow, indicating a gel-like clot. Additional PBM sessions were given for active bleeding or sluggish oozing, with assessments every 5 min over a total observation period of 30 min. The assessment duration was 10 s. The time to achieve hemostasis was recorded upon clinical assessment of complete clot formation, based on 100% agreement between the operator and the researcher (ST). Supplementary hemostasis measures were applied as needed based on the operator’s decision.

### 2.6. Routine Dental Treatments in This Study

Tooth extraction, SRP or minor oral surgery were performed by the experienced dentist at each center. Routine tooth extractions were carried out using standardized and atraumatic techniques [13]. SRP involved ultrasonic supragingival scaling and root planing (Woodpecker DTE D600 LED Ultrasonic Piezo Scaler, Guilin Woodpecker Medical Instrument Co., Ltd., Guilin, Guangxi, China), followed by subgingival hand scaling (Paradise Dental Technologies, Missoula, MT, USA). Local anesthesia was administered if needed. Pre-session PBM prior to SRP was recommended for active periodontal diseases with severe inflamed gingivae. Soft tissue surgeries, including gingivectomy, frenectomy and incision, were performed using laser surgery. Conventional periodontal crown lengthening was also undertaken, followed by additional PBM initiating blood clot formation. For postoperative instruction, analgesics were prescribed every 6 h as needed. Patients followed a soft diet for 2 days and were instructed to contact emergency services nearby or contact researcher if postoperative bleeding occurred. Follow-up evaluations were scheduled for 2 to 4 weeks if not possible, a phone interview was conducted by the operator at each center.

### 2.7. Data Collection

The demographic and clinical data were recorded using data recording form. Intraoral photographs were taken during the preoperative, perioperative, postoperative, and follow-up periods using a DSLR Canon 90D with a Canon EF 100 mm f/2.8 L macro lens (Canon, Melville, NY, USA). The data gathering was conducted by ST. The cut-off point for hemostasis, defined as achieving hemostasis within 5 min after employing laser-mediated techniques, was determined and assessed by the operator for each case reviewed by ST.

### 2.8. Assessment of Hemostasis Achieved and Wound Healing

Based on anonymized clinical data and photographs (2.7), the data validation was evaluated by the operators (SPS, KM, STT and SS) and the data collector (ST). Clinical hemostasis achieved was assessed based on the following criteria: (i) positive: more effective or faster bleeding control comparing to non-laser therapy, (ii) neutral: hemostasis achieved at a rate comparable to normal hemostasis, (iii) negative: hemostasis slower than usual, (iv) partial effectiveness: postoperative bleeding occurred 2 to 3 h after treatment and (v) non-effectiveness: bleeding continued beyond 12 h, requiring return visits. The wound healing assessment was made by each operator and based on the anonymized before and after dental treatment photographs. The criteria were as followed: (i) recovery: complete healing or minimal inflammation, (ii) positive partial recovery: faster or better than normal healing, (iii) neutral partial recovery: healing comparable to normal, (iv) negative partial recovery: delayed healing compared to standard treatment and (v) no-response: incomplete healing.

Postoperative bleeding is defined as returning to the clinic due to active bleeding that persists beyond 12 h after treatment or any delayed bleeding from the extraction wound within 4 weeks. Clinical complications included any unexpected adverse events such as infection, delayed wound healing or patient discomfort requiring additional intervention. All adverse events were assessed during the follow-up period or via phone interview and recorded using a data recording form.

### 2.9. Variable and Measurement

The primary outcome of this study was the clinical efficacy of laser-mediated hemostasis, defined as hemostasis achieved within 5 min, as assessed by the operator at each research site. Additional clinical outcomes included wound healing, incidence of postoperative bleeding and clinical complications.

### 2.10. Statistical Analysis

To demonstrate the overall clinical outcome of laser-mediated hemostasis in assisting routine dental treatments, the clinical data were pooled for statistical analyses. The normal distribution regarding ages and the number of laser irradiations were explored using Shapiro-Wilk tests. The demographic characteristics of included patients as well as the general characteristics of extraction sockets per sockets, SRP and minor oral surgery per procedures were demonstrated. Continuous data were described as averages with 95% confidence intervals (95% CI), while categorical data were presented as frequencies. The Chi-Square test was used to compare patient proportions across three different dental treatments, with Fisher’s exact test applied when expected frequencies were low. Subgroup analyses were conducted to compare the patient proportion across three different dental treatments and other clinical outcomes. Data was processed and analyzed using SPSS (version 29.0.2.0, IBM Corp., Armonk, NY, USA). All analyses were two-sided with a significant level set at 0.05.

## 3. Results

### 3.1. Demographic Data

The study included 38 females and 22 males across three research networks. Half of these patients had systemic diseases, 17 were classified as ASA-PS III or IV and 16 were undergoing oral antithrombotic therapy. Thirty-three of the 60 patients received routine dental treatments at a secondary-level healthcare unit. Regarding the pooled data, the average age was 63.35 years old, with a 95% confidence interval of 61.06 to 65.64 years. Based on the types of routine dental treatments, Two-thirds underwent tooth extraction or SRP, while the remaining received minor oral surgery or combined treatments. The demographic characteristics of the patients included in the study are presented in Table 2.

### 3.2. Clinical Data

All routine dental treatments were categorized into three groups, namely, tooth extraction, SRP and minor oral surgery. Each treatment was counted per extraction socket or per procedures. There were 94 extraction sockets and 28 procedures of SRP. Additionally, 18 minor oral surgery procedures were conducted, including 6 crown lengthening procedures, 5 frenectomy procedures, 3 gingivectomy procedures and 4 oral soft tissue surgeries. The number of extraction sockets performed in patients classified as ASA PS III and IV was significantly higher compared to those receiving SRP and minor oral surgery (*p* < 0.001). The number of patients on antithrombotic medication who received tooth extraction was higher than the SRP and minor oral surgery groups (*p* = 0.039). Out of the extracted teeth, 55 were removed due to periodontal disease and 39 due to pulp necrosis or pulpitis. The severity of gingival inflammation was also significantly greater in the extraction procedure compared to the others (*p* < 0.001). Across three dental procedures, laser therapy was predominantly used for local hemostasis following routine dental treatments (*p* = 0.054).

In the context of laser-mediated hemostasis techniques (Table 3 and Table 4), the combination of HILT for ablating sulcular fibers and PBM for initiating blood clot formation proved significantly effective for achieving hemostasis in extraction sockets. Conversely, PBM alone was the preferred hemostasis technique following SRP and minor oral surgeries (*p* < 0.001). According to operator experience, laser-mediated hemostasis provided more rapid control of bleeding than conventional hemostatic methods in 97.86% of procedures, with no significant differences noted among the various dental procedures (*p* = 0.701). No cases of postoperative bleeding were reported. Furthermore, 61.43% of procedures achieved hemostasis within five minutes, again with no significant differences across the dental procedures (*p* = 0.092).

Further analysis showed that moderate to severe gingival inflammation significantly prolonged the duration of hemostasis following both tooth extractions and SRP (*p* < 0.001). During the follow-up period, the outcomes of wound healing were generally favorable. Although four extraction sockets were excluded from the analysis due to participant dropout, the healing observed in the laser-assisted tooth extraction group was significantly greater than in the other groups (*p* = 0.005). The study results consistently demonstrated the clinical efficacy of laser-mediated hemostasis and the associated wound healing index across all procedures.

## 4. Discussion

Preventing postoperative bleeding in older patients undergoing routine dental treatments is a significant challenge for dental professionals. Achieving hemostasis during these surgical procedures is essential, especially for older patients who are on oral antithrombotic therapy, have multiple systemic diseases or suffer from untreated periodontal disease. These conditions significantly raise the risk of excessive bleeding during and after dental treatments. In this study, we accepted the null hypothesis, demonstrating that laser-mediated hemostasis was equally effective across various routine dental procedures, as indicated by achieving hemostasis within 5 min.

This study highlighted the clinical effectiveness of laser-mediated hemostasis in reducing bleeding and promoting blood clot formation in older patients undergoing routine dental procedures. The use of laser therapy for hemostasis significantly reduced the time needed for bleeding control per socket in older patients who underwent tooth extraction [12]. In this study, HILT was used to ablate sulcular fibers for controlling soft tissue bleeding. Meanwhile, PBM was applied to enhance blood clot formation after tooth extraction, SRP and minor oral surgery procedures.

Klangthong et al. (2022) [10] suggested that PBM-enhanced blood clot formation through focal aggregation of red blood cells (RBC) at the superficial surface of the bleeding socket. Hemoglobin molecules absorbed laser energy, which subsequently induced localized RBC aggregation, thereby facilitating physiological coagulation and clot formation [10]. Previous clinical studies supported the benefits of PBM initiating blood clot formation by reducing the time to achieve hemostasis [1,10,11,14,15] without any clinical complications [16,17].

There was no instances of postoperative bleeding and clinical complications in all three settings. This emphasizes the safety and clinical effectiveness of laser-mediated hemostatic techniques. These techniques will be beneficial for geriatric patients who are at high risk of bleeding during dental treatment, even in a primary care setting. Operators preferred a combination of HILT and PBM for tooth extraction to achieve optimal hemostatic outcomes, while PBM alone was utilized for SRP and minor oral surgeries. The results likely reflected the operators’ selection of hemostatic techniques based on perceived bleeding risk, the ease of use and the practical advantages of laser-mediated hemostasis techniques.

A novel PBM initiating blood clot formation in periodontal therapy has demonstrated clinical efficacy. This study indicated that inflamed gingivae significantly prolonged the duration of hemostasis during simple tooth extractions and SRP. The findings align with Cheng et al., which reported that gingival inflammation impairs both hemostasis and wound healing by enhancing vascular permeability and the inflammatory response [18]. Most SRP procedures related to inflamed gingivae required 5 to 15 min to achieve hemostasis. This study recommends preoperative PBM for one week prior to SRP to reduce gingival inflammation. Moreover, PBM with an energy density of at least 4 J/cm^2^ and a power output below 500 mW has shown clinical efficacy in reducing postoperative pain and enhanced healing when used in conjunction with periodontal treatment or surgery [19,20].

In addition to provide clinical hemostatic benefits, laser therapy has demonstrated an ability to enhance oral wound healing. PBM using red or infrared lasers promotes healing by activating transforming growth factor-beta 1 (TGF-1) [21,22], platelet-derived growth factor (PDGF) and interleukin-8 (IL-8) [23], which played key roles in re-epithelialization, angiogenesis and anti-inflammatory responses [21,22,23,24]. It was well-established that tissue injury induced the release of inflammatory mediators, leading to cell damage and pain during the inflammatory phase [25]. Previous studies have shown that PBM can reduce inflammation and pain while also accelerating the healing of wounds [26,27,28,29].

The results of this study support the clinical application of combining HILT with the ablation of sulcular fibers and/or PBM in routine dental practices, including tooth extraction, SRP, and minor oral surgeries. These methods are safe and effective, especially for geriatric patients, including those undergoing antithrombotic therapy. These techniques are easily applicable across all levels of the healthcare system, including primary care.

To enhance the reliability and reproducibility of clinical findings in future research, it is essential to utilize objective outcome measures. Further clinical trials should investigate the effectiveness of laser-mediated hemostasis in periodontal surgery, particularly for addressing bleeding from both bone and soft tissue in older patients who have a tendency to bleed due to medication or multiple underlying health conditions. Additionally, basic scientific research is necessary to clarify the mechanisms by which PBM contributes to hemostasis.

## 5. Conclusions

Based on criteria of this study, a combination of HILT ablating sulcular fiber and PBM initiating blood clot formation effectively reduced hemostatic duration, prevented postoperative bleeding and promoted healing of the extraction socket. Additionally, PBM alone also showed effective hemostatic capabilities following SRP and minor oral surgery in older patients undergoing routine dental treatments.

## Figures and Tables

**Table 1:** Laser operating parameters used in this study.

Parameter (Units)	PBM Initiating Blood Clot Formation		HILT Ablating Soft Tissue Loosening	
Wavelength	635 nm	810 ± 10 nm	810 ± 10 nm	980 nm
Laser machine model and manufacturer	SmartM, Lasotronix, Piaseczno, Poland	PICASSO^TM^ LITE, AMD Lasers, Indiana	PICASSO^TM^ LITE, AMD Lasers, Indiana	SmartM, Lasotronix, Piaseczno, Poland
Laser type	diode, semiconductor	Gallium-aluminum- arsenide (GaAlAs), diode laser	Gallium- Aluminum- arsenide (GaAlAs), diode laser	diode, semiconductor
Actual power output	0.2 W	0.8 W	2.5 W	3 W
Energy density (J/cm^2^)	5.08	5.93	-	-
Diameter of optic delivery probe	8 mm	6 mm	320 micron	320 micron
Fiber initiation	-	-	required	not required
Emission mode	continuous wave			
Technique employed	contact mode, stationary, perpendicular to the extraction socket		contact mode, moving up and down into the periodontal sulcus	
Approximate treatment time	10 s per session	5 s per session	-	-

**Table 2:** Demographic data in this study.

Demographic Data	Number of Subjects (n = 60)
**Research networks**	
Bua Ngoen promoting hospital	14
Borabue hospital	33
Dental hospital, Khon Kaen University	13
**Gender**	
Male	22
Female	38
**Average age**	63.35
Range of age (years) 50 to 87	50 to 87
**ASA physical status**	
ASA-PS I, II	43
ASA-PS III, IV	17
**Patient health conditions (n = 30)**	
Cardiovascular diseases	14
Other systemic diseases	16
**Taking oral antithrombotic therapy (n = 16)**	
SAPT (aspirin)	8
DAPT	3
Warfarin	5
**Type of routine dental treatment**	
Tooth extraction	21
Scaling and root planing	19
Minor oral surgery	13
Combined dental treatments	7
**Type of tooth extraction (n = 28)**	
Single	12
Multiple	16
**Type of scaling and root planing (n = 24)**	
By quadrant	8
Full mouth	16
**Type of minor oral surgery (n = 14)**	
Periodontal surgery; crown lengthening and	7
gingivectomy	
Oral soft tissue surgery; frenectomy, biopsy, incision etc.	7

n—number of subjects; ASA-PS—American Society of Anesthesiologists Physical Status; SAPT—single antiplatelet therapy; DAPT—dual antiplatelet therapy.

**Table 3:** Results of clinical outcome assessments: Utilization of laser-mediated hemostasis and its effectiveness.

Outcome Measurements	Tooth Extraction (94 Sockets)	Scaling and Root Planing (28 Procedures)	Minor Oral Surgery (18 Procedures)	*p* Value
**Type of laser-mediated hemostasis**				
PBM (47.86%)	27	27	13	<0.001 ^a^
PBM+HILT (52.14%)	67	1	5	
**Time to achieved hemostasis**				
Within 5 min (61.43%)	59	13	14	0.092 ^a^
Between 5 to 30 min (38.57%)	35	15	4	
**Effectiveness of laser-mediated hemostasis**				
Positive hemostasis achieved (97.86%)	92	27	18	0.701 ^a^
Neutral hemostasis achieved (2.14%)	2	1	0	
Negative hemostasis achieved (0)	0	0	0	
Partial effective (0)	0	0	0	
Non-effective (0)	0	0	0	
**Postoperative bleeding**	0	0	0	-
**Wound healing index**				
Recovery (93.57%)	90	25	16	0.005 ^a^
Positive partial recovery (3.57%)	0	3	2	
Neutral partial recovery (0)	0	0	0	
Negative partial recovery (0)	0	0	0	
Non-response (0)	0	0	0	
**Loss follow-up (2.86%)**	4	0	0	-

PBM—photobiomodulation; HILT—high-intensity laser therapy; ^a^ Fisher exact test.

**Table 4:** Subgroup analyses by procedures.

Dental Treatments	Clinical Outcomes (per Sockets or per Procedures)	Time to Achieved Hemostasis			Effectiveness of Laser- Mediated Hemostasis			Wound Healing Index		
		5 mins	5 to 30 mins	*p* Value	Hemostasis Achieved	Partial or Non- Effective	*p* Value	Recovery or Positive Partial Recovery	Non- Response	*p* Value
**Tooth** **extraction**	**(a) Oral antithrombotic therapy**									
	With (29)	18	11	0.926 ^b^	29	0	-	26	0	-
	Without (65)	41	24		65	0		64	0	
	**(b) Gingival inflammation level**									
	Mild (67)	53	14	<0.001 ^b^	67	0	-	63	0	-
	Moderate to severe (27)	6	21		27	0		27	0	
	**(c) Type of laser-mediated hemostasis**									
	PBM (27)	24	3	<0.001 ^a^	27	0	-	27	0	-
	PBM+HILT (67)	35	32		67	0		63	0	
**Scaling and root planing**	**(a) Oral antithrombotic therapy**									
	With (3)	2	1	0.583 ^a^	3	0	-	3	0	-
	Without (25)	11	14		25	0		25	0	
	**(b) Gingival inflammation level**									
	Mild (9)	9	0	<0.001 ^a^	9	0	-	9	0	-
	Moderate to severe (19)	4	15		19	0		19	0	
	**(c) Type of laser-mediated hemostasis**									
	PBM (27)	13	14	1.000 ^a^	27	0	-	27	0	-
	PBM+HILT (1)	0	1		1	0		1	0	
Minor oral surgery	**(a) Oral antithrombotic therapy**									
	With (2)	2	0	1.000 ^a^	2	0	-	2	0	-
	Without (16)	12	4		16	0		16	0	
	**(b) Type of laser-mediated hemostasis**									
	PBM (13)	10	3	1.000 ^a^	13	0	-	13	0	-
	PBM+HILT (5)	4	1		5	0		5	0	

PBM—photobiomodulation; HILT—high-intensity laser therapy; ^a^ Fisher exact test; ^b^ Chi-Square test.

## Data Availability

The data presented in this study are available on request from the corresponding author.
